# Cognitive and motor reserve in Parkinson's disease: Two sides of the same coin?

**DOI:** 10.1111/jnp.70056

**Published:** 2026-06-02

**Authors:** Isabella Anzuino, Sonia Di Tella, Alice Tondinelli, Clio Scopetani Testa, Martina Petracca, Maria Rita Lo Monaco, Anna Rita Bentivoglio, Maria Caterina Silveri

**Affiliations:** ^1^ Department of Psychology Catholic University of the Sacred Heart Milan Italy; ^2^ Movement Disorders Unit Fondazione Policlinico Universitario ‘Agostino Gemelli’ IRCCS Rome Italy; ^3^ Institute of Internal Medicine and Geriatrics Catholic University of the Sacred Heart Rome Italy; ^4^ Center for the Medicine of Aging Fondazione Policlinico Universitario ‘Agostino Gemelli’ IRCCS Rome Italy; ^5^ Institute of Neurology Catholic University of the Sacred Heart Rome Italy; ^6^ Present address: Experimental and Applied Psychology Laboratory, Department of Health and Life Sciences European University of Rome Rome Italy

**Keywords:** cognitive functions, cognitive reserve, motor functions, motor reserve, Parkinson's disease

## Abstract

Cognitive reserve (CR) and motor reserve (MR) are constructs that can explain why some people are more resilient than others to neurodegenerative diseases such as Parkinson's disease (PD). However, it is unclear whether these reserves exert domain‐specific or cross‐domain influences. This study investigated the predictive role of CR and MR on cognition and motor function, as well as possible cross‐domain interactions. Fifty individuals with PD underwent neuropsychological and motor assessments and completed questionnaires measuring CR (Cognitive Reserve Index questionnaire, CRIq), MR (Motor Reserve Index questionnaire, MRIq), and current physical activity (Current Physical Activity questionnaire, CPAq). Multiple regression models were computed to assess the predictive value of total reserve indices on cognition, executive functions, and clinical motor outcomes. Correlation analyses were conducted between reserve sub‐indices and cognitive and clinical motor variables. Total CRIq scores predicted global cognitive functioning and executive abilities, as well as age of onset and disease duration. However, total MRIq scores did not demonstrate any predictive value. The CPAq total score predicted executive functions and disease stage. Some CRIq and MRIq sub‐indices were found to correlate with both cognitive and motor measures. CR influences multiple domains in PD, supporting cognition and counteracting clinical decline. The MRIq total score did not demonstrate any predictive value, but some correlations emerged between MRIq sub‐indices and cognitive and motor measures. The CPAq revealed effects that crossed domains, indicating that ongoing physical activity may confer immediate protective effects. This highlights the importance of promoting cognitive and physical stimulating lifestyles to enhance resilience in PD.

## INTRODUCTION

The rapid ageing of the global population has made cognitive impairment in adults a common clinical sign of neurodegenerative diseases. Investigating factors that enable some people to age more successfully than others is important for improving quality of life and guiding interventions to delay or mitigate cognitive decline.

The concept of ‘reserve’ (Stern, 2002) was introduced to describe the brain's ability to resiliently cope with neurological damage or normal ageing. Cognitive reserve (CR) is the best‐known type, and it is proposed to account for the discrepancy between brain damage and clinical manifestations (Stern et al., 2020, 2023). CR refers to resources accumulated by engaging in stimulating intellectual activities over time (Stern, 2009). Proxies of CR include education, intelligence quotient, and occupational complexity. The hypothesis is that cognitive engagement can change the brain and improve neural plasticity (Alvares Pereira et al., 2022).

Parkinson's disease (PD), the second most common neurodegenerative disorder, is characterized by progressive degeneration of dopaminergic neurons, resulting in reduced dopamine levels (Postuma et al., 2015). The disease manifests a wide range of motor and non‐motor symptoms, which become evident since the prodromal stage.

Research on PD highlights the role of CR in supporting cognitive performance, particularly in executive functions (Ciccarelli et al., 2018), and potentially mitigating cognitive decline (Barulli & Stern, 2013; Hindle et al., 2014, 2016). Evidence suggests CR also positively impacts motor functions in PD (Kotagal et al., 2015), particularly in individuals with higher education levels or cognitively stimulating lifestyles (Blume et al., 2017; Jeong et al., 2022), protecting motor abilities across various disease stages (Guzzetti et al., 2019) and possibly preserving basal ganglia functionality (Di Tella et al., 2023).

Motor reserve (MR) is an emerging construct representing an individual's capacity to maintain motor functionality despite neuropathology (Bastos & Barbosa, 2022). Since PD is characterized by motor dysfunction due to dopaminergic striatal depletion, MR may help explain discrepancies between the severity of parkinsonian motor symptoms and the observed dopaminergic deficit (Chung et al., 2020; Sunwoo et al., 2017). The ‘residual‐based approach’ is the conventional way to quantify MR. This method compares observed and predicted motor scores on the Unified Parkinson's Disease Rating Scale (UPDRS), considering age, disease duration, and dopamine transporter activity in the posterior putamen (Chung et al., 2020). However, this approach might oversimplify MR (Bastos & Barbosa, 2022), which is also influenced by physical activity. The hypothesis that the effects of physical activity throughout the lifespan may accumulate, thereby increasing resilience to motor ability decline, is sustained by evidence that exercise promotes neuroplastic changes, which in turn support motor function in both ageing and pathological conditions (Bastos & Barbosa, 2022; Hoenig et al., 2023). The MR score can be obtained by measuring an individual's motor engagement, defined here as the active and habitual involvement in different physical activities throughout life. This includes both deliberate exercise (e.g., playing sports) and incidental physical activity (e.g., walking for transportation, domestic chores or physically demanding work) (Pucci et al., 2024). The literature robustly supports that, in healthy ageing, a physically active lifestyle plays a crucial role in maintaining or enhancing cognitive performance, particularly executive function (Liu‐Ambrose, 2010), processing speed, and memory (Audiffren & André, 2019). Chung and colleagues (Chung et al., 2022) conducted a study to investigate the association between MR and cognition in PD, and the findings revealed positive correlations between MR and global cognitive function, as well as the domain of verbal memory. Bai et al. (2024) confirmed the association between MR and both motor and non‐motor functions in PD, finding that patients with different levels of MR exhibited varying rates of motor and cognitive symptom progression. PD patients who exhibited lower MR showed an elevated risk of postural instability and progression to mild cognitive impairment or dementia. This suggests that patients with higher MR may be more capable of managing brain damage than those with lower MR, providing evidence that MR can offer resilience not only to motor but also cognitive deterioration (Bai et al., 2024). Research on other movement disorders, such as spinocerebellar ataxia type 2 (SCA2), confirms that MR proxies measured via functional brain network preservation correlate with less severe motor symptoms and better cognition, with physical activity during premorbid periods contributing to MR (Siciliano et al., 2022). Furthermore, a novel study on adult and elderly healthy populations revealed that the most influential factors on cognitive performance were age and CR, but also physical activity across the lifespan (i.e., MR proxy) and current physical activity (CPA), with a stronger effect on executive functions (Pucci et al., 2024).

In summary, although some studies indicate a specific protective value of CR and MR on cognitive and motor function, respectively (Chung et al., 2020; Ciccarelli et al., 2018; Sunwoo et al., 2017), many studies suggest a more general protective value of both reserves on both cognition and movement (Guzzetti et al., 2019; Siciliano et al., 2022). Conventionally, the domains of cognition and motor function are considered distinct. However, recent research shows the neural networks underlying both are closely interconnected (Hoenig et al., 2023), and both motor structures (e.g., basal ganglia) and non‐motor structures (e.g., ventral striatum, orbitofrontal cortex) are involved in contrasting functional decay in PD (Chung et al., 2020; Shine et al., 2019). The question of whether distinguishing between CR and MR remains unclear. It is possible they contribute independently or rely on common mechanisms.

This study aimed to evaluate the effects of CR and MR in counteracting motor and cognitive decline in people with PD, with a specific focus on executive functions. This choice is founded on empirical evidence that executive processes are among the earliest and most consistently impaired cognitive domains in PD, reflecting fronto‐striatal dysfunction and representing a sensitive marker of both disease progression and compensatory mechanisms. Specifically, the study investigates whether these two forms of reserve exert domain‐specific effects—CR on cognition and MR on movement—or whether they provide cross‐domain protective effects. In addition to the MR score, the CPA index (Pucci et al., 2024) was also provided to investigate whether MR, resulting from the accumulation of positive effects from physical activity throughout life, could be distinguished from the beneficial effects of physical activity ‘per se’ at the time of assessment. Observing people with PD, a disease that affects cognitive and motor control systems, provides a unique opportunity to directly compare the protective effects of CR and MR on cognition and movement.

## MATERIALS AND METHODS

### Subjects and clinical evaluation

Fifty PD patients in mild‐to‐moderate stages were recruited from the Day Hospital of Geriatrics and the Movement Disorders Outpatient Clinic of Agostino Gemelli IRCCS University Polyclinic Foundation, Rome, Italy. Inclusion criteria included: over 18 years of age; idiopathic PD diagnosis according to Movement Disorder Society criteria (Postuma et al., 2015); a minimum of 5 years of education; stable therapy for at least 2 months or drug‐naïve; Modified Hoehn & Yahr (H&Y) scale ≤3 (Goetz et al., 2004; Postuma et al., 2015); UPDRS ≤33 (Martínez‐Martín et al., 2015). Exclusion criteria comprised neurological comorbidities unrelated to PD, severe psychiatric disorders, serious systemic conditions, and poor compliance. Patients with a diagnosis of dementia were excluded from the study. Mild leukoencephalopathy was not exclusionary.

Patients underwent neurological evaluation and detailed clinical history assessment. Both H&Y and UPDRS III scores were employed as indicators of clinical severity. While the UPDRS III provides a detailed quantitative assessment of motor symptoms, the H&Y scale captures global disease staging, thus allowing a more comprehensive characterization of motor function impairment. Moreover, functional independence in performing daily activities was assessed using the Schwab and England Activities of Daily Living (SEADL) scale (Schwab & England, 1969). This scale provides a percentage score reflecting the patient's ability to perform daily tasks, with higher scores indicating greater autonomy. Current therapy was quantified via levodopa equivalent daily dose (LEDD). The cognitive assessment included general functioning, as measured by the Montreal Cognitive Assessment (MoCA) (Dapor et al., 2025), and executive functions, as measured by the Word Fluency Test (WFT) (Carlesimo et al., 1996), the Digit Span Backward test (Monaco et al., 2013), the Stroop test (Caffarra et al., 2002), the Key Search test (Wilson et al., 1996), and the Cognitive Estimation Task (CET) (Della Sala et al., 2003). The estimation of premorbid intelligence was conducted using the Brief Intelligence Test (originally named *‘Test di Intelligenza Breve’*—TIB) (Colombo et al., 2000).

### Assessment of cognitive and motor reserve

We used the 20‐item Cognitive Reserve Index questionnaire (CRIq) to measure CR (Nucci et al., 2012). The CRIq is a retrospective tool that captures cognitively stimulating activities over the lifespan (from the age of 18). It allows for the calculation of a total score (CRIq_Tot), expressed on a scale with a mean of 100 and a standard deviation of 15. According to established thresholds, an elevated CRIq is indicative of a higher estimated CR. The questionnaire also permits the calculation of three sub‐indices: CRI‐Education (CRIq_Edu) based on education and training courses; CRI‐Working Activity (CRIq_WA), based on work responsibilities; CRI‐Leisure Time (CRIq_LA), referring to activities that require cognitive effort. The three subscores and the total index of CR were calculated using an Excel file for automated calculation, which was made freely available by the authors who developed the questionnaire.

The MR was assessed using the Motor Reserve Index questionnaire (MRIq) (Pucci et al., 2024). It is a 17‐item questionnaire designed to estimate the cumulative MR acquired through physical activity across the lifespan (since the age of 18 years). The MRIq is divided into six‐section subscores, each covering specific activities: domestic (MRIq_DA), walking (MRIq_Walk), leisure (MRIq_LA), physical exercise (MRIq_PE), caring (MRIq_CA), and workplace (MRIq_WA). An Excel spreadsheet enables automated calculation of the total score (MRIq_Tot). MRIq_Tot is expressed as the percentage of time engaged in physically demanding activities relative to the individual's age. It is computed by dividing the frequency measured in terms of years spent performing the activity by the total age (from 18 years to the age at the time of assessment), and multiplying the result by 100.

Another instrument was utilized to evaluate CPA levels. This questionnaire, designated as the Current Physical Activity questionnaire (CPAq) (Pucci et al., 2024), encompasses 17 items and is analogous to the MRIq, although focusing exclusively on the previous 12 months. Four alternatives can be selected for each item based on weekly frequency: never, rarely (once a week), sometimes (two/three times a week), and often (more than three times a week). The CPAq is divided into different areas in the same way as the MRIq and provides the same six‐section subscore. The overall CPAq score (CPAq_Tot) is derived by summing the scores of the individual items (range 0–51).

### Statistical analyses

Statistical analyses were conducted using SPSS (version 29 IBM SPSS, Armonk, NY, USA) and JASP (version 0.19.3). Continuous variables had means and standard deviations, while categorical variables had frequencies and percentages. Skewness, kurtosis, and the Shapiro–Wilk test were used to check the distribution of the data. Pearson's r or Spearman's ρ, as needed, were used to determine the correlation method. If bivariate normality was not met, Spearman's was used instead. Correlation coefficients were calculated for the CRIq, MRIq, and CPAq total scores. Subsequently, correlations between each of these indices and age were computed. To investigate the impact of CR, MR, and CPA on cognitive performance and clinical motor characteristics, multiple linear regression models were conducted. Age was included as a predictor variable. The rationale for including age was twofold: first, because age is known to influence cognitive performance (Pucci et al., 2024), and second, because it is associated with a reduction in daily physical activity (Teraž et al., 2022).

#### Effect of reserve indices on cognition

To assess the effect of CR and MR on overall cognitive functioning, two multiple linear regression models were developed with the MoCA as the dependent variable. In the first model, MRIq was included among the predictors (MRIq model) (other predictors: age and CRIq), while in the second model, MRIq was replaced by CPAq (CPAq model) (other predictors: age and CRIq) to address the potential issue of collinearity between MR and CPA. By using the same sets of predictors as above, multivariate multiple regression models were conducted to specifically evaluate the effects of CR and MR on executive functioning. The dependent variables in these models included the scores on executive function tasks: Digit Span Backward, WFT, Stroop test, Key Search test, and CET. Due to the integration of education (a primary proxy for CR) into the CRIq measure, the analyses were conducted with the raw scores.

#### Effects of reserve indices on motor function

In addition, the effect of MRIq and CPAq, including age and CRIq as predictors for each model, was evaluated on PD motor symptom severity (as measured by the UPDRS and H&Y scales), levodopa dosage (LEDD), disease duration and age at symptom onset, using multivariate multiple regression models.

#### Association between reserve sub‐indices and cognitive and motor domains

Finally, exploratory correlational analyses were performed to determine the relationships between subscores of CRIq, MRIq, and CPAq and the patients' performance in cognitive tasks and motor and clinical variables.

## RESULTS

### Clinical and neuropsychological characterization of the recruited sample

Table 1 provides a comprehensive overview of the demographic, clinical, and neuropsychological data collected in the PD sample. As illustrated, the examined subjects exhibited sustained global cognitive functionality and consistent average performance across all domains of assessment. Specifically, the mean scores for both general screening and specialized executive tasks fell consistently within the normal clinical range, thus confirming that the sample consists of cognitively preserved PD patients without dementia. A detailed analysis of individual performances revealed that 1 out of 50 participants (2%) scored below the normative cut‐off on the MoCA. With regard to the executive battery, the proportion of participants who performed below the normative threshold was also low: two patients (4%) for the Digit Span Backward, one patient (2%) for the WFT, and four patients (8%) for the Stroop test—errors. A greater frequency of performance falling below the established normative values was exclusively observed for the CET, with 12 patients (24%) achieving scores above the cut‐off for bizarre responses.

**TABLE 1:** Overview of PD patients characteristics.

*Socio‐demographic characteristics*	
Age, mean (SD)	66.14 (8.78)
Education, mean (SD)	13.58 (3.91)
Male gender, *n* (%)	33 (66.00)
*PD motor screening*	
H & Y, median (IQR)	2.00 (.00)
UPDRS III, mean (SD)	18.92 (7.06)
SEADL, mean (SD)	84 (14)
LEDD, mean (SD)	624.40 (273.56)
Onset age, mean (SD)	58.33 (8.12)
Disease duration, years, mean (SD)	7.80 (3.92)
*Neuropsychological assessment*	
MoCA, mean (SD) [cut‐off > 17.95]	24.74 (3.19)^a^
Digit Span Backward, mean (SD) [cut‐off > 2.65]	4.49 (1.40)^a^
WFT, mean (SD) [cut‐off ≥ 17.35]	37.45 (12.73)^a^
Stroop test—errors, mean (SD) [cut‐off ≤ 4.23]	.84 (1.98)^a^
Key Search test, mean (SD)	6.79 (3.19)
CET—bizarreness, mean (SD) [cut‐off < 4.00]	3.33 (2.00)^a^
TIB, mean (SD)	111.44 (9.30)
*Reserve questionnaires*	
CRIq_Tot, mean (SD)	123.00 (19.88)
MRIq_Tot, mean (SD)	40.08 (14.86)
CPAq_Tot, mean (SD)	15.34 (5.89)

Abbreviations: CET, Cognitive Estimation Task; CPAq, Current Physical Activity questionnaire; CRIq, Cognitive Reserve Index questionnaire; H&Y, Hoehn and Yahr Scale; IQR, interquartile range; LEDD, Levodopa Equivalent Daily Dose; MoCA, Montreal Cognitive Assessment Test; MRIq, Motor Reserve Index questionnaire; *n*, frequencies; PD, Parkinson's disease; SD, standard deviation; SEADL, Schwab and England Activities of Daily Living scale; TIB, Brief Intelligence Test; UPDRS III, Unified Parkinson's Disease Rating Scale motor part III; WFT, Word Fluency Test.

^a^ Adjusted score for age and education according to normative data (cut‐off in brackets).

### Correlation between CRIq, MRIq and CPAq scores, and age

The correlational analysis performed using Pearson's test revealed a substantial correlation between MRIq_Tot and CPAq_Tot (Pearson's *r* = .590, *p* < .001). No significant correlations were found between CRIq_Tot and MRIq_Tot, nor between CRIq_Tot and CPAq_Tot (*p* > .050). A significant negative correlation between age and CPAq_Tot was also observed (Pearson's *r* = −.451, *p* = .001), with older participants reporting less engagement in physical activity at present, consistent with an age‐related decline in exercise. In contrast, no significant correlations were detected between age and CRIq_Tot, nor between age and MRIq_Tot (*p* > .050).

### Predicting value of age, CRIq, MRIq and CPAq on cognitive performance

The results from the regression analyses are reported below. With regard to overall cognitive functioning, in the first model (MRIq model), with MoCA as the dependent variable, two of the three predictors proved to be statistically significant: age (*β* = −.554, *p* < .001), with younger participants showing higher MoCA scores, and CRIq_Tot (*β* = .353, *p* = .006), with higher CR levels associated with better performance. In the second model (CPAq model), age (*β* = −.484, *p* < .001) and CRIq_Tot (*β* = .306, *p* = .016) were found to remain significant predictors. However, both MRIq_Tot and CPAq_Tot exhibited a lack of statistical significance in their respective models. The impact of reserve indices on executive functioning was further examined through multivariate multiple regression models, predicting performance across a battery of executive function tasks. Post hoc univariate analyses were conducted to explore the specific contribution of CRIq_Tot to each executive measure. CRIq_Tot consistently emerged as a significant predictor within these multivariate models (MRIq model: Wilks' Λ = .658, *F*(5, 39) = 4.06, *p* = .005; CPAq model: Wilks' Λ = .651, *F*(5, 39) = 4.18, *p* = .004), highlighting its key role in supporting executive functions in PD. Specifically, CRIq_Tot was a significant positive predictor of Digit Span Backward in the first model (MRIq model: *β* = .023, *p* = .045) and of verbal fluency, as measured by the WFT, in both the MRIq and CPAq models (MRIq model: *β* = .243, *p* = .020; CPAq model: *β* = .231, *p* = .021). For the Key Search test, CRIq_Tot maintained significant effects (MRIq model: *β* = .063, *p* = .012; CPAq model: *β* = .055, *p* = .023). Furthermore, higher CRIq_Tot scores were significantly associated with better performance on the CET, reflected in fewer bizarre responses (MRIq model: *β* = −.042, *p* = .004; CPAq model: *β* = −.042, *p* = .002). In contrast, MRIq_Tot and CPAq_Tot did not demonstrate significant predictive effects in these models, with the exception of CPAq_Tot, which emerged as a significant predictor of CET—bizarreness (CPAq model: *β* = −.101, *p* = .039). Age also contributed significantly in both models, particularly influencing inhibitory control as measured by the Stroop test (MRIq model: *β* = .122, *p* = .005; CPAq model: *β* = .117, *p* = .012), with older patients showing more errors, consistent with an age‐related decline in inhibitory control. Furthermore, age was also found to be a significant predictor of performance on the Key Search test and the CET in the MRIq model (Key Search test: *β* = −.123, *p* = .033; CET—bizarreness: *β* = .071, *p* = .038), with younger patients exhibiting better results across these measures. For further details, refer to Tables 2 and 3.

**TABLE 2:** Model fit measures (*R* ^2^ and *p*‐value) and model coefficients (standardized *β* and *p*‐value) of regression analysis with age, CRIq and MRIq as predictors (MRIq model) and cognitive function test scores and clinical motor characteristics as dependent variables.

	Model fit measures	Predictors *β* (*p*)		
	*R* ^2^ (*p*)	Age	CRIq_Tot	MRIq_Tot
*Global cognitive functioning*				
MoCA	.354 (**<.001**)	−.554 (**<.001**)	.353 (.**006**)	−.148 (.233)
*Executive functioning tasks*				
Digit Span Backward	.103 (.192)	−.033 (.222)	.023 (.**045**)	−.015 (.307)
WFT	.126 (.118)	−.231 (.333)	.243 (.**020**)	−.049 (.709)
Stroop test—errors	.177 (.**037**)	.122 (.**005**)	−.026 (.155)	.015 (.506)
Key Search test	.181 (.**034**)	−.123 (.**033**)	.063 (.**012**)	−.028 (.374)
CET—bizarreness	.261 (.**004**)	.071 (.**038**)	−.042 (.**004**)	−.015 (.413)
*Clinical motor characteristics*				
UPDRS III	.006 (.968)	−.029 (.826)	.006 (.922)	.028 (.713)
H&Y	.087 (.247)	.008 (.293)	−.001 (.813)	−.006 (.150)
LEDD	.043 (.571)	6.404 (.198)	−1.868 (.385)	1.101 (.699)
Onset age	.822 (**<.001**)	.809 (**<.001**)	.074 (.**009**)	−.027 (.456)
Disease duration	.236 (.**007**)	.186 (.**005**)	−.080 (.**005**)	.029 (.433)

*Note*: *p*‐values < .050 are highlighted in bold.

Abbreviations: CET, Cognitive Estimation Task; CRIq, Cognitive Reserve Index questionnaire; H&Y, Hoehn and Yahr Scale; LEDD, Levodopa Equivalent Daily Dose; MoCA, Montreal Cognitive Assessment Test; MRIq, Motor Reserve Index questionnaire; UPDRS III, Unified Parkinson's Disease Rating Scale—motor part III; WFT, Word Fluency Test.

**TABLE 3:** Model fit measures (*R* ^2^ and *p*‐value) and model coefficients (standardized *β* and *p*‐value) of regression analysis with age, CRIq and CPAq as predictors (CPAq model) and cognitive function test scores and clinical motor characteristics as dependent variables.

	Model fit measures	Predictors *β* (*p*)		
	*R* ^2^ (*p*)	Age	CRIq_Tot	CPAq_Tot
*Global cognitive functioning*				
MoCA	.342 (**<.001**)	−.484 (**<.001**)	.306 (.**016**)	.106 (.438)
*Executive functioning tasks*				
Digit Span Backward	.081 (.299)	−.027 (.352)	.020 (.080)	.002 (.951)
WFT	.123 (.126)	−.210 (.408)	.231 (.**021**)	.013 (.970)
Stroop test—errors	.169 (.**045**)	.117 (.**012**)	−.022 (.205)	.000 (.994)
Key Search test	.171 (.**043**)	−.102 (.098)	.055 (.**023**)	.043 (.609)
CET—bizarreness	.321 (**<.001**)	.047 (.168)	−.042 (.**002**)	−.101 (.**039**)
*Clinical motor characteristics*				
UPDRS III	.008 (.950)	−.068 (.630)	.015 (.790)	−.096 (.633)
H&Y	.214 (.**012**)	.000 (.979)	−.001 (.713)	−.033 (.**003**)
LEDD	.062 (.409)	3.690 (.486)	−1.391 (.498)	−7.636 (.313)
Onset age	.823 (**<.001**)	.848 (**<.001**)	.065 (.**017**)	.094 (.336)
Disease duration	.237 (.**006**)	.150 (.**033**)	−.071 (.**010**)	−.082 (.406)

*Note*: *p*‐values < .050 are highlighted in bold.

Abbreviations: CET, Cognitive Estimation Task; CPAq, Current Physical Activity questionnaire; CRIq, Cognitive Reserve Index questionnaire; H&Y, Hoehn and Yahr Scale; LEDD, Levodopa Equivalent Daily Dose; MoCA, Montreal Cognitive Assessment Test; UPDRS III, Unified Parkinson's Disease Rating Scale—motor part III; WFT, Word Fluency Test.

### Predicting value of age, CRIq, MRIq and CPAq on clinical motor characteristics

CR and MR measures did not significantly predict clinical markers of disease severity, including UPDRS III scores, H&Y scale, and LEDD. However, in the CPAq model, post hoc univariate analyses revealed that CPAq_Tot is a significant negative predictor of H&Y stage (*β* = −.033, *p* = .003), with higher levels of CPA being associated with less severe disease. Notably, CRIq_Tot emerged as a strong and consistent predictor of both age at disease onset and disease duration across both models. Specifically, higher CRIq_Tot scores were found to be significantly associated with a later onset of motor symptoms (MRIq model: *β* = .074, *p* = .009; CPAq model: *β* = .065, *p* = .017) and with a shorter disease duration (MRIq model: *β* = −.080, *p* = .005; CPAq model: *β* = −.071, *p* = .010). Similarly, age emerged as a significant predictor of age at onset in both models (MRIq: *β* = .809, *p* < .001; CPAq: *β* = .848, *p* < .001), as well as of disease duration (MRIq: *β* = .186, *p* = .005; CPAq: *β* = .150, *p* = .033). See Tables 2 and 3 for further information.

### Correlation between sub‐indices of CRIq, MRIq and CPAq and clinical variables

Positive correlations emerged between CRIq_WA and WFT (rho = .340, *p* = .016) and Key Search test (rho = .367, *p* = .011) and between CRIq_LA and WFT (*r* = .323, *p* = .022). Additionally, a negative correlation was identified between CRIq_LA and the CET—bizarreness score (rho = −.405, *p* = .004). CRIq_Edu and CRIq_LA were significantly correlated with the age of onset of motor symptoms (*r* = .397, *p* = .005 and *r* = .294, *p* = .040, respectively). Furthermore, CRIq_WA showed a negative association with disease duration (rho = −.329, *p* = .021). A positive correlation was detected between the MRIq_PE subscore and global cognitive functioning, as measured by the MoCA test (rho = .299, *p* = .035). Additionally, the CPAq_PE subscore was found to be significantly associated with the MoCA (rho = .356, *p* = .011) and the WFT (rho = .371, *p* = .008). Significant negative correlations emerged between the MRIq_PE subscore and disease duration (rho = −.371, *p* = .009) and between the MRIq_WA subscore and severity of motor symptoms, as evaluated by the UPDRS III scale (*r* = −.311, *p* = .028). A trend was observed between the MRIq_WA sub‐index and disease stage on the H&Y scale (rho = −.277, *p* = .051). Finally, the analysis revealed negative correlations between CPAq_LA and H&Y disease stage (rho = −.306, *p* = .031) and between CPAq_PE and the H&Y scale (rho = −.420, *p* = .002), as well as between CPAq_PE and disease duration (rho = −.551, *p* < .001).

## DISCUSSION

The importance of maintaining cognitively and physically active lifestyles to promote healthy ageing has been increasingly recognized. The central question that this study seeks to address is the degree to which individuals' pre‐existing cognitive and motor abilities contribute to their capacity to cope with neurodegenerative processes. Despite the existence of a substantial body of research examining CR, only a limited number of studies have explored the specific impact of MR. Consequently, there is a gap in our understanding of MR's role in preserving brain health. The present study provides compelling evidence for the differential contributions of CR, MR, and CPA to cognitive and motor functioning in PD.

We observed that CR exerts a beneficial influence across different domains, as expected. More precisely, CR, measured by the CRIq, significantly predicted global cognitive performance and executive functioning. Higher scores on the CRIq scale were found to be predictive of better performance across a variety of executive tasks, including those involving working memory, verbal fluency, planning, and cognitive estimation. Furthermore, CRIq was identified as a robust predictor of both the onset and duration of the disease. In more detail, it was found that higher CRIq scores were predictive of a delayed age at the onset of symptoms and consequently a shorter duration of the disease. These regression results were complemented by correlation analyses with CRIq subcomponents, which provided further insights into the specific contributions of different life experiences. This is consistent with previous studies that indicated CR acts as a buffer against cognitive decline (Ciccarelli et al., 2018) by enhancing neural efficiency and compensatory mechanisms (Barulli & Stern, 2013; Stern et al., 2020). In relation to the predictive function of CR on the basal ganglia and dorsolateral‐prefrontal circuitry, an MRI study by Di Tella and colleagues (Di Tella et al., 2023) revealed that CR proxies can contribute to the preservation of the structural integrity of these critical regions, which are indicative of the brain status of PD. The finding that CR also predicted clinical motor variables, such as the age of motor symptoms onset and disease duration, extends prior evidence, suggesting that cognitive enrichment across the lifespan may modulate not only cognitive resilience but also disease expression at the motor level. By showing that higher levels of sustained cognitive engagement over time are effectively associated with a later onset of motor symptoms, this study contributes to the conceptualization of CR beyond a simple ‘cognitive buffer’ to a potential modulator of the clinical expression and natural trajectory of the disease. However, it is important to interpret these hypothesized ‘cross‐domain’ effects with caution. While CR significantly predicted the timing of disease onset and disease duration, it did not reveal a significant relationship with current motor severity, as measured by UPDRS III or H&Y scores. Consequently, while the impact of CR is clearly evident in the cognitive domain and in the timeliness of disease manifestation, its role in conferring direct motor resilience against current motor disability appears more limited.

MR, measured using MRIq, did not emerge as a significant predictor of cognitive or motor outcomes. A preliminary evaluation suggests a discrepancy between this result and the conclusions of previous studies that indicated an association between MR and the long‐term prognosis of PD (Bai et al., 2024). However, exploratory correlation analysis suggested MR subcomponents may contribute to resilience mechanisms both in cognition and in the clinical motor domain. This is consistent with previous research suggesting that distinct aspects of MR may underpin protective effects in patients diagnosed with SCA2 (Siciliano et al., 2022).

The present study contributes to the existing literature by providing a more integrated and multilayered perspective on resilience in PD. Contrary to the prevailing trend in the field of research, which has predominantly examined CR in isolation, our models adopt a more comprehensive approach by considering the accumulation of reserve indices over time, including both cognitive and motor‐related proxies, together with current lifestyle engagement. This methodological paradigm facilitates the examination of the independent contributions of trait‐like, developmentally accumulated factors and more proximal, state‐dependent behavioural engagement within the same analytical framework. In this context, CPA can be conceptualized as an additional correlate of cognitive and functional outcomes, alongside traditional indicators of reserve. This distinction is of crucial significance, since it suggests that resilience in PD is not a static ‘endowment’ built solely in the past, but rather a dynamic process where ongoing physical exercise offers immediate, cross‐domain benefits to executive functions and functional status, regardless of prior habits. Importantly, this pioneering study was the first to attempt to estimate MR in a sample of PD patients using an ad hoc MR questionnaire. Several factors may account for the absence of significant effects of MRIq in the regression models. First, MR is a relatively novel construct that has not been as thoroughly theorized as CR (Stern et al., 2020, 2023). Second, in contrast to CR, which is supported by well‐established proxies such as education, MR is characterized by the absence of standardized metrics and theoretical consensus. For the purposes of this study, we employed a retrospective questionnaire to estimate cumulative physical activity. Despite its innovative nature, the instrument shares the intrinsic limitations of self‐report measures, including susceptibility to recall bias. This issue may be of particular relevance to MRIq, which requires the recollection of activities performed several decades earlier, whereas CRIq includes some more objectively verifiable components (e.g. years of schooling). The heterogeneity in the operationalization of physical activity across the lifespan—ranging from incidental domestic tasks to deliberate physical exercise—renders it challenging for a retrospective instrument such as the MRIq to capture a unified index of motor enrichment. Finally, beyond measurement and conceptual issues, it can be hypothesized that the characteristics of the cohort under investigation also played a role. The participants were non‐demented PD patients in the mild‐to‐moderate stage, with limited motor impairment. In such cases, the degree of motor dysfunction may not have been sufficiently robust to ‘challenge’ reserve mechanisms, thereby masking the potential effects of MR.

It is noteworthy that CPA, as evaluated through CPAq, exhibited cross‐domain associations, indicating that ongoing engagement in physical activity, independent of lifetime cumulative exposure, yields quantifiable benefits on cognition and functional status. Significantly, CPAq focuses on more recent behavioural patterns (within the past 12 months), which may be less influenced by long‐term recall difficulties and consequently may be reported with greater reliability. This aligns with the ‘virtuous cycle’ model by Audiffren and André (Audiffren & André, 2019), which posits that regular physical activity enhances executive function by modulating brain plasticity and neurotrophic signalling, facilitating the maintenance of healthy behaviours. Within this theoretical framework, the distinction between lifelong reserve accumulation and current habitual behaviour is congruent with the Brain Maintenance paradigm, which proposes that daily active lifestyle choices may mitigate age‐ or disease‐related cerebral changes (Stern et al., 2020). CPAq captures ongoing, habitual activity, which can exert direct effects on neuroplasticity, functional connectivity, and day‐to‐day performance over time. Conversely, MRIq provides a retrospective, cumulative estimate that may be less sensitive to mild disease. This interpretation matches previous findings that regular physical activity can improve cognitive function and slow the progression of motor symptoms in PD, even when initiated in late adulthood (Petzinger et al., 2013). Furthermore, it is in accordance with extant longitudinal data that suggests maintaining regular exercise is more impactful in slowing PD progression than the level of activity at the time of diagnosis (Tsukita et al., 2022).

From a clinical perspective, this supports the hypothesis that the Parkinsonian brain remains highly responsive to behavioural changes, thus reinforcing the ‘never too late’ message for initiating neuroprotective lifestyle interventions.

## LIMITATIONS

Despite the original contributions of this study, several limitations should be acknowledged. The relatively modest sample size may compromise the generalizability of the findings and the statistical power to detect more subtle effects, particularly with regard to the MRIq. Moreover, the cross‐sectional design precludes any inference about temporal relationships between reserve proxies and clinical outcomes, which necessitates confirmation through longitudinal studies. Methodological constraints related to measurement should also be considered. Although the MR's status as an innovative construct is already recognized, it is currently lacking in the extensive theoretical and metric standardization that has already been established for the CR framework. In the final analysis, the absence of neuroanatomical correlates, such as neuroimaging data (e.g., MRI or PET), or molecular biomarkers, limits the possibility of directly linking these behavioural reserve indices to specific underlying brain mechanisms or compensatory networks. It is imperative that future research integrates these biological measures to validate the structural and functional basis of the reserve constructs in PD.

## CONCLUSIONS

Taken together, these observations underscore the importance of distinguishing between lifelong reserve‐building processes and the immediate effects of current behaviours. They also highlight the need to encourage mentally and physically stimulating lifestyles to improve the resilience and quality of life of individuals with PD. Future research should refine MR measurement tools and explore the dynamic interplay between CR, MR, and CPA across different stages of PD. Exploration of the specific cerebral circuits implicated in both CR and MR in PD remains a topic that requires further investigation. Furthermore, the analysis of molecular biomarkers may facilitate the elucidation of the biological basis of reserve in neurodegenerative conditions.

## AUTHOR CONTRIBUTIONS

**Isabella Anzuino:** Project administration; data curation; investigation; formal analysis; writing – original draft. **Sonia Di Tella:** Project administration; methodology; formal analysis; writing – review and editing. **Alice Tondinelli:** Data curation; investigation; writing – review and editing. **Clio Scopetani Testa:** Data curation; investigation; writing – review and editing. **Martina Petracca:** Data curation; methodology; writing – review and editing. **Maria Rita Lo Monaco:** Data curation; methodology; writing – review and editing. **Anna Rita Bentivoglio:** Supervision; writing – review and editing. **Maria Caterina Silveri:** Conceptualization; funding acquisition; supervision; project administration; writing – review and editing.

## FUNDING INFORMATION

The study was funded by the PRIN programme of the Italian Ministry of University and Research (Ministero dell'Università e della Ricerca), Protocol No. 2022H2J3N3.

## CONFLICT OF INTEREST STATEMENT

The authors declare that they have no known competing financial interests or personal relationships that could have appeared to influence the work reported in this paper.

## ETHICS STATEMENT

The study was approved by the Lazio Area 3 Ethics Committee, Protocol No. 0000805/24, dated 24 April 2024. In order to participate in the study, all individuals were required to sign an informed consent form.

## Data Availability

The data that support the findings of this study are available from the corresponding author upon reasonable request.
